# The role of androgens in experimental rodent mammary carcinogenesis

**DOI:** 10.1186/s13058-014-0483-x

**Published:** 2014-11-25

**Authors:** Jaesung Choi, Basil Psarommatis, Yan Ru Gao, Yu Zheng, David J Handelsman, Ulla Simanainen

**Affiliations:** ANZAC Research Institute, University of Sydney, Sydney, 2139 NSW Australia

## Abstract

Breast cancer is currently the most frequent, fatal cancer of women in western countries. While estrogens have a widely understood involvement in breast cancer, a significant but not yet fully understood role for androgens has also been suggested. The principal androgen, testosterone, is the obligate steroidal precursor of estradiol, but can equally be metabolized into dihydrotestosterone, a more potent, pure androgen. Both androgens exert their distinctive biological effects via the androgen receptor, which is coexpressed with estrogen receptor alpha in 80 to 90% of breast cancers. The hormonal control of breast development and pathology has been examined experimentally through the use of animal models, notably mice and rats. This review summarizes the data from experimental rodent models on the effects of androgens in experimental breast cancer, aiming to address the importance of androgens and the androgen receptor in the origins and pathogenesis of breast cancers, as well as to discuss potential biomarker and therapeutic opportunities arising from novel insights based on the experimental research.

## Introduction

Breast cancer is currently among the most frequent and fatal cancers afflicting women worldwide. The strongest epidemiological clues to the hormonal origins of breast cancer arise from the recognition of lifetime endogenous estrogen exposure comprising early menarche, incessant ovulation, and late first lactation and menopause. While estrogens have a widely understood involvement, we [1] and others (reviewed in [2]) have demonstrated a significant but not yet fully understood role for androgens in breast cancer. The hormonal control of breast development and pathology has been examined through animal model experimentation, most frequently involving mice and rats.

This review summarizes the experimental data using rodent models on the effects of androgens in experimental breast cancer, aiming to highlight the role of androgens and androgen receptor (AR)-mediated androgen effects in experimental breast cancers. In addition, the review aims to lay a solid foundation for a consensus, as well as guidance for future research into novel biomarkers and therapeutic targets arising from novel insights acquired from experimental research. Owing to increased interest in androgen actions in breast cancer, we will cover the historical aspects briefly, but concentrate on the most recent literature focusing on the role of androgens in experimental rodent breast tumorigenesis. This focus on experimental animal models aims to provide a complementary view to recent reviews of androgens and breast cancer, which concentrate on clinical data and human breast cancer cell lines [2],[3].

## Androgens, androgen receptor and breast cancer

### Androgens and the androgen receptor

Androgens are a group of 19-carbon steroid hormones produced mainly in the testes, but to a lesser extent in other steroidogenic tissues such as ovaries, adrenal glands, and placenta, as well as in peripheral tissues, including adipose tissue and mammary glands. Dehydroepiandrosterone (DHEA) and androstenedione are pro-androgens (androgen precursors) capable of being converted into testosterone and/or dihydrotestosterone (DHT) in peripheral and androgen target tissues. The major circulating androgen, testosterone, either can be aromatized (via aromatase enzyme) into estradiol (E_2_) acting via estrogen receptor (ER) signaling or can be reduced (via 5?-reductases) within target tissues to the nonaromatizable androgen DHT, which has higher androgenic bioactivity than testosterone and is associated with a higher affinity for AR [4], leading to more potent androgen signaling. While DHT is a nonaromatizable androgen, it can be further reduced irreversibly to 3?-androstanediol that may activate ER? [5].

Androgens (testosterone, DHT) and pro-androgens (DHEA, androstenedione) are the most abundant sex hormones produced in women, with the normal ovary producing larger amounts of androgens than E_2_; however, E_2_ is two orders of magnitude more potent on a molar basis than testosterone or DHT. Testosterone serves as an obligate precursor for E_2_ synthesis, so androgens play an indirect but necessary role in female physiology. Yet ARs are also expressed in virtually every tissue in women, including breast tissue, suggesting a direct physiological role for the AR-mediated androgen effects [6].

### Evidence for the role of androgens in human breast cancer

Significant roles for androgens in human breast cancer susceptibility and as treatment options have received more attention recently. The AR is expressed in 70 to 90% of breast cancers, comparable with ER? (70 to 80%) and progesterone receptor (50 to 70%) positivity. Yet AR-negative breast cancers respond poorly to hormone therapy with reduced overall survival, while conversely AR-positive cancers are smaller with fewer lymph node metastases corresponding to a better prognosis, thus demonstrating the role of the AR as a biomarker [7]. Importantly, AR expression is detected in about 10 to 50% of triple-negative (ER, progesterone receptor and Her-2) breast cancers that respond poorly to traditional therapies (reviewed in [3]). Similarly, natural models of high endogenous androgen exposure ? such as women suffering from polycystic ovarian syndrome and congenital adrenal hyperplasia, as well as men with much lower risk of breast cancer when compared with women ? support the concept that androgen action may protect against breast cancer [8],[9].

## Rodent breast cancer models

The history of experimental breast cancer has been reviewed extensively [10], and therefore our aim is to concisely describe the models used to explore the role of androgens in experimental rodent breast cancers.

### Chemical carcinogenesis

One of the most remarkable findings in breast cancer research is the ability to induce breast-specific tumors in rodents, usually not susceptible to spontaneous breast cancer, using chemical carcinogens [11]. Common molecular mechanisms that possibly underlie chemical carcinogenesis involve activating mutations of ras proto-oncogenes [12]. Interestingly, the activation of ras proto-oncogenes following chemical carcinogenesis could be regulated by other signaling pathways. This hypothesis was demonstrated by a lack of H-ras-activating mutations and upregulation at the expression level following 7,12-dimethylbenz(a)anthracene (DMBA)-induced skin carcinogenesis in PTEN knockout mice [13]. Similarly, androgens acting via AR could regulate ras proto-oncogene activation in chemical carcinogenesis as supported in hormonally induced prostate cancer [14]. Androgens could therefore modify carcinogen-induced mammary tumors either by directly regulating proliferation and cell cycle via targeting growth factors or by the regulation of underlying molecular mechanisms in chemical carcinogenesis.

#### Dimethylbenz(a)anthracene and methylcholanthrene

Most early research examining the role of androgens in carcinogen-induced experimental breast cancer used methylcholanthrene (MCA) [15],[16]. But DMBA has become the current mainstay because only a single dose is required to induce mammary tumors in 100% of female rats with a mean time of 95?days (reviewed in [17]). DMBA and MCA are polycyclic aromatic hydrocarbons that act through the aryl hydrocarbon receptor. They are also pro-carcinogens requiring cytochrome P450 (1A1, 1A2 and 1B1) activation to become carcinogens and subsequently form metabolite-DNA adducts. Aryl hydrocarbon receptor activation transcriptionally regulates cytochrome P450 (CYP) enzymes, but can also act directly by regulating cell growth, apoptosis, and transition to an invasive, metastatic phenotype [18]. Aryl hydrocarbon receptor activation and inducible CYP systems are therefore central to chemical carcinogenesis induced by polycyclic aromatic hydrocarbons, a process that may be susceptible to regulation by androgens [19],[20]. DMBA treatment also induces ovarian damage characterized by small afollicular ovaries and reduced circulating androgens [1],[21], but with increased circulating follicle-stimulating hormone and luteinizing hormone [1]. This damage is more prominent in mice than rats, which may contribute to mice having a greater susceptibility to DMBA-induced mammary tumors.

#### *N*-Nitroso-*N*-methylurea

In contrast to DMBA and MCA, *N*-nitroso-*N*-methylurea is a direct-acting carcinogen that does not require metabolic activation. This carcinogen can be advantageous in experiments that involve co-treatments or genetic modification, which may influence CYP activation [16].

### Hormonal carcinogenesis

Spontaneous breast cancer is very rare in rodents and therefore hormonal treatments are required to induce breast cancers (early work extensively reviewed in [22]). Long-term continuous exposure to exogenous E_2_ or the pro-estrogen estrone (E_1_) in rats induces a high incidence of mammary tumors that frequently metastasize. Tumor latency depends on the estrogen dose as well as the rat strain and age [22]. The mechanisms suggested for estrogen-induced carcinogenicity include activation of ER signaling pathways [23] or the metabolism of estrogen into oxidative metabolites having genotoxic, mutagenic, transforming, and carcinogenic effects [24]. Androgen actions could therefore either modify ER-dependent signaling [25] or the metabolism of E_2_ and E_1_ by P450 enzymes [26]. Administration of either natural (progesterone) or synthetic (medroxyprogesterone acetate) progestins increases the carcinogen-induced mammary tumor incidence in mice [27]. Medroxyprogesterone acetate may function as a promoter for DMBA-induced carcinogenesis or by increasing the incidence of DMBA-induced mutations involving the H-ras proto-oncogene [27]. However, it is important to note that medroxyprogesterone acetate is also a potent activator of AR [28].

### Spontaneous carcinogenesis

Some specific rodent strains are highly prone to spontaneous mammary tumor formation. Spontaneous mammary tumors occur over a range from 40% for Fischer rats to over 80% for Buff/N rats [29]. Similarly the C3H mouse strain with a high lifetime incidence (>70%) of mammary tumors has been used to study androgen effects on mammary tumors [30]. Mammary tumors in C3H mice are mainly due to the presence of mouse mammary tumor viruses that induce tumors by acting as insertional mutagens, which in turn activates the expression of cellular proto-oncogenes. Viral transcription is regulated by specific sequences within the long terminal repeat that are also responsive to androgen action [31] and could modify the initiation of tumors, but cannot explain the androgen effect on existing tumors. Rat and mouse lines differing in their sensitivity to spontaneous mammary tumors have been used to determine genetic components, including dominant resistance genes as well as sensitivity genes that modify susceptibility to spontaneous and/or induced mammary tumors and could be shared by human tumors.

### Genetically modified mouse models

The development of genetically modified, transgenic or knockout, mouse models has greatly advanced our understanding of the molecular basis of initiation, promotion, and progression of breast cancer. Numerous genetic mouse models used in breast cancer studies include APC, BRCA1, ERBB2 (Neu), p53, and PTEN knockout mouse models (reviewed in [10]). Mammary gland-specific, targeted inactivation of relevant genes such as the *Ar* (Cre-loxP using promoters targeting mammary cells; Table?1) combined with breast cancer models are promising approaches to investigate androgenic regulation of specific pathways and effects on mammary gland pathology [32].

**Table 1:** **Promoters driving transgene expression in mammary cells**

Promoter	Origin	Expression
MMTV-LTR	Mouse mammary tumor virus	Breast epithelial cells, several other tissues
WAP	Whey acidic protein	Secretory mammary epithelium
C3(1)	Rat prostate steroid-binding protein	Epithelial cells of prostate and mammary gland
B-LG	Bovine ?-lactoglobulin	Mammary gland
MT	Metallothionein	Most mammary cells

### Relevance of rodent breast cancer models to human breast cancer

Rodent models are irreplaceable for exploring the *in vivo* biology and mechanisms of tumor initiation, promotion, progression, and metastasis. These models provide unique help to identify otherwise unrecognized opportunities for novel treatment and diagnostic or prognostic markers through experiments not feasible in humans. Mammary tumors in rodents and humans display many etiological similarities. In both species, spontaneous tumors develop late in life and with low frequency, while genetic factors increase both the susceptibility and frequency of but reduce the latency of overt tumors ([33]; see Androgens and spontaneous rodent carcinogenesis). Furthermore, hormonal induction of breast cancer is directly proven in rodent models and is a well-accepted inference in human breast cancers from large observational studies of estrogen-treated women [34]. Similarly, carcinogen-induced experimental breast cancer is proven in rodents (for example, polycyclic aromatic hydrocarbons) and is suspected (see Androgens and chemical carcinogenesis) but less clearly established in human breast cancers. While no single rodent model can address all aspects of human breast cancer, different experimental models provide complementary findings to decipher the heterogeneity of established but fragmentary pathophysiological knowledge currently available. The evaluation of experimental breast cancer model(s), while quite germane to human breast cancer, must therefore consider critically the appropriate caveats on the design and interpretation of findings for such species extrapolation.

Different experimental breast cancer models have distinct features that should be considered in choosing an optimal model for the study of interest. Considerations should include histological similarities as well as the degree of genetic or transcriptomic similarity (including steroid hormone receptor expression and hormone sensitivity). Although it is desirable to use similar morphological descriptors to describe human and rodent mammary tumors, the morphology of rodent tumors is quite distinctive and trained pathologists would not confuse them with human tumors [35]. For example, the main histotypes in DMBA-induced mouse mammary tumors are carcinomas/adenocarcinomas or squamous cell carcinomas [1], while the latter are very rare in women [36]. On the other hand, spontaneous tumors tend to have hyperproliferation of both connective and glandular tissues and are generally classified as fibroadenomas [37].

Interestingly, while mammary tumor development in rodents and women is highly estrogen dependent [38], rat mammary tumors are mainly estrogen responsive whereas mouse mammary tumors are mainly estrogen independent, with human breast cancers falling between rat and mouse tumors with regard to estrogen sensitivity [38]. To integrate the estrogen-independent mammary tumors in mice into a more estrogen-sensitive framework, specific ER-positive mammary tumor mouse models have been developed (references cited in [39]) and we found that DMBA generates both ER?-positive and ER?-negative tumors in mice [1]. These ER-positive models could therefore be used to test the possible modifying effect of androgens on ER?-dependent effects in breast cancer [25].

The transcriptomic similarity of human breast cancers and experimental mammary tumors in mouse models has been analyzed recently [39]. Where some models developed tumors with model-specific gene expression and histopathological patterns (for example, TgC3(1)-*Tag* model) suitable for specific mechanistic studies, others had greater diversity of tumors (for example, DMBA) [39]. Like human breast cancers, mouse mammary tumors can also be divided into basal or luminal types of clusters ? although luminal tumors with estrogen responsiveness and ER? positivity are less common in mice compared with women [39].

Rodent breast cancer models have the advantage of being able to produce a wide variety of breast cancer subtypes that are observed in breast cancer patients. Hence, using a judicious choice of rodent models allows researchers to investigate different subtypes of breast cancer by creating specific opportunities as a platform to study underlying biological mechanisms of mammary carcinogenesis, which cannot be directly investigated in humans. With the appropriate design of experiments as well as careful selection of experimental models, rodent models allow the identification of basic processes in breast cancer development, but also the means to test the roles of specific pathways as well as potential preventive and curative agents.

## Effects of androgens in rodent breast cancer models

### Androgens and chemical carcinogenesis

Chemical carcinogens, notably DMBA, MCA, and *N*-nitroso-*N*-methylurea, have been widely used in experimental studies to explore the role of androgens in breast cancer since the mid twentieth century (Tables?2, 3 and 4). The chemical carcinogen studies are categorized into androgen effects on tumor incidence (Tables?2 and 3) or regression (Table?4) as discussed below.

**Table 2:** **Androgen effects on tumor incidence (induced by chemical carcinogens)**

Reference	Animal model	Type of androgen	Dose ^a^	Duration	Incidence
**7,12-Dimethylbenz(a)anthracene**					
Briziarelli [40]	SD rats^b^	TP	0.01, 0.05, 0.5, 1 or 1.5?mg daily	30?days	? (at doses of 0.5 to 1.5?mg)^c^
Briziarelli [41]	SD rats	TP	0.13, 0.26, 0.4 or 10.5?mg daily	10?days	? (0.26 only)
Li and colleagues [42]	SD rats	DHEA	(Not described)	Implant	?
Luo and colleagues [43]	SD rats	DHEA	5, 10 or 20?mg daily	9?months	?
Kohama and colleagues [44]	Wistar rats	DHEA or TE	150?mg/kg DHEA or 60?mg/kg TE weekly	22?weeks	?
**Methylcholanthrene**					
Shay and colleagues [15]	Wistar rats	T	37 or 75?mg	Implant	?
Shay and colleagues [45]	Wistar rats	T	75?mg	Implant	?
***N*** **-Nitroso-** ***N*** **-methylurea**					
Lubet and colleagues [46]	SD rats	DHEA	5, 24, 120, 600 or 2,000?mg/kg DHEA in diet	109?days	? (600/2,000)

DHEA, dehydroepiandrosterone; SD, Sprague?Dawley; T, testosterone; TE, testosterone enanthate; TP, testosterone propionate. ^a^Some doses are shown as weekly doses calculated based on the dosing regime in the original article. ^b^Female rats unless otherwise specified. ^c^When TP treatment started 10 or 20?days after 7,12-dimethylbenz(a)anthracene but not later.

**Table 3:** **Neonatal androgen effects on tumor incidence (induced by chemical carcinogens)**

Reference	Animal model	Type of androgen	Dose (mg)	Duration	Incidence
**7,12-Dimethylbenz(a)anthracene**					
Kov?cs [52]	Wistar rats^a^	TP	2.5	Single injection	?
Shellabarger and Soo [53]	SD rats	TP	1.25	Single injection	?
Christakos and colleagues [54]	SD rats	TP	1.25	Single injection	?
Yoshida and colleagues [55]	SD rats	TP	1.25	Daily until the end of the experiment	?
Purnell [56]	LEW/Mai rats	TP	1.25	Single injection	No effect
Verhoeven and colleagues [57]	Female and male SD rats	TP	0.5	Single injection	No effect

SD, Sprague?Dawley; TP, testosterone propionate. ^a^Female rats unless otherwise specified.

**Table 4:** **Androgen effects on tumor growth (induced by chemical carcinogens)**

Reference	Animal model	Type of androgen	Dose ^a^	Tumor growth
**7,12-Dimethylbenz(a)anthracene (using androgens)**				
Young and colleagues [60]	SD rats^b^	T	1.2 to 6?mg weekly	?
		DHT	1.2 to 6?mg weekly	
Heise and Gorlich [61]	SD rats	TP	6 to 180?mg/kg weekly	? (low dose only)
Teller and colleagues [62]	SD rats	MDTP and TP	Total of 480?mg for 8?weeks	?
Shimkin and colleagues [16]	SD/Wistar rats	DP	200?mg weekly for 6?weeks	No effect
Teller and colleagues [63]	SD rats	MDTP and TP	3.75 to 60?mg weekly for 8?weeks	?
Teller and colleagues [64]	SD rats	MDTP	60?mg weekly for 8?weeks	?
Mobbs [67]	SD rats	TP	10 or 50?mg/kg weekly or a single 60?mg pellet	? (high dose only)
Griswold and Green [68]	SD rats	MDTP and TP	10?mg/kg daily for 20?days	?
Briziarelli and colleagues [69]	SD rats	FBTA	2 or 4?mg daily for 30?days	?
Takahashi and colleagues [70]	SD rats	MDTP and TP	6?mg weekly	?
Quadri and colleagues [71]	SD rats	DmP	0.5?mg daily for 3?weeks	?
Horn and colleagues [72]	Inbred rats	Calsterone	10?mg daily for 2 to 3?weeks	?
Costlow and colleagues [73]	SD rats	TP	8?mg weekly	?
Zava and McGuire [74]	SD rats	TP	2.4?mg weekly	?
Teller and colleagues [65]	SD rats	MDTP	200?mg/kg weekly for 5?weeks	?
Teller and colleagues [66]	SD rats	MDTP	3 to 100?mg/kg weekly for 4?weeks	? (dose dependent)
Dauvois and colleagues [51]	SD rats	DHT	3?cm silastic implant (30/100, DHT/cholesterol, w/w)	?
Boccuzzi and colleagues [75]	SD rats	DHEA	4?mg daily for 21?days	?
Boccuzzi and colleagues [76]	SD rats	DHT	25??g DHT daily for 20?days	?
Gatto and colleagues [77]	SD rats	DHEA	4?mg daily for 20?days	?
**Methylcholanthrene (using androgens)**				
Ercoli and Briziarelli [79]	SD rats	DHT or DHT enol ethers	2?mg daily for 30?days	?
Shimkin and colleagues [16]	SD/Wistar rats	DP	200?mg/kg weekly for 6?weeks	No effect
**7,12-Dimethylbenz(a)anthracene (using anti-androgens)**				
Spinola and colleagues [78]	SD rats	Flutamide	10?mg daily for 20?days	?
Boccuzzi and colleagues [76]	SD rats	Flutamide	2?mg daily for 20?days	?

DHEA, dehydroepiandrosterone; DHT, dihydrotestosterone; DmP, dromostanolone propionate; DP, drostanolone propionate; FBTA, 9?-fluoro-11?-hydroxybenzo[d,e] testosterone 17-acetate; MDTP, 2?-methyl-DHT propionate; SD, Sprague?Dawley; T, testosterone; TP, testosterone propionate. ^a^Some doses are shown as weekly doses calculated based on the dosing regime in the original article. ^b^Female rats unless otherwise specified.

#### Androgen effects on tumor incidence

Studies have shown (Tables?2 and 3) that androgen treatment of rodents before the appearance of palpable DMBA, MCA, or *N*-nitroso-*N*-methylurea-induced tumors significantly decreased mammary tumor incidence [15],[40]-[46] and increased latency [40],[46].

Androgens (testosterone propionate (TP), unesterified testosterone, DHT) or pro-androgens (DHEA) were generally administered by subcutaneous injection around the time of chemical carcinogen treatment, with the duration of androgen treatment varying from single injection to continuous treatment until tumor(s) developed. As noted previously (see Androgens and the androgen receptor), some androgens can be enzymatically modified to other potent bioactive steroids such as the aromatization of testosterone into E_2_ and the 5?-reduction to DHT, while other androgens may be metabolized to corresponding estrogens or activated or inactivated by 5?-reduction. Similarly, DHT can be reduced to 3-diols [47], while the pro-androgen DHEA can be metabolized to testosterone, DHT or E_2_. Nevertheless, despite different androgens being used in these experiments, all of the studies reported that androgens reduced chemically induced mammary tumor incidence (Table?2) and increased tumor latency at certain doses of androgen treatment. These findings imply that the strong, AR-mediated androgen effect on tumor incidence in carcinogen-induced mammary tumors could be via AR-dependent regulation of cell proliferation and/or CYP-mediated carcinogen activation and formation of DNA adducts as discussed in Chemical carcinogenesis.

The mechanism(s) for androgen-induced inhibition of chemically induced mammary carcinoma still remains unclear. Androgens may inhibit pituitary and ovarian function [48]; however, androgens could also have direct effects because rodent mammary epithelium as well as carcinogen-induced mammary tumors are AR-positive [49],[50]. This latter interpretation of direct effects is supported by the fact that potent DHT inhibition of the growth of DMBA-induced mammary tumors persists in ovariectomized rats [51].

DMBA-induced tumor incidence has also been examined after neonatal androgenization of female rats (Table?3) [52]-[57], with androgens first injected between 0 to 5?days after birth and treatment varying from a single injection to continuous treatment until the end of the experiment. Most studies [52]-[55], but not all [56],[57], found that, like peri-pubertal treatments, neonatal androgenization reduced tumor incidence. The protective role of neonatal androgenization could be due to depressed pituitary and ovarian function following neonatal androgen treatment [53],[54] or reduced P4 levels in sexually mature females that could modify the response to carcinogens [55]. This hypothesis is supported by the finding that P4 treatment of neonatally androgenized females increased the DMBA-induced mammary cancer incidence [58]. In addition, neonatal androgenization causes lactational differentiation of the mammary epithelium [52],[59] that may modify responses to DMBA.

DMBA alone does not induce mammary tumors in Sprague?Dawley male rats, whereas when E_2_ is co-administered 50% develop tumors within 26?weeks [38]. This is comparable with our findings in male mice where DMBA caused enlargement of mammary lymph nodes, but not the growth of mammary tumors in either wild-type or AR knockout males for up to 39?weeks [1]. These findings demonstrate that exposure to ovarian hormones, notably the sole bioactive estrogen E_2_, is necessary to promote carcinogen-induced rodent mammary tumors.

#### Androgen effects on tumor growth

Most studies [51],[60]-[79], but not all [16], found that androgen treatment after the appearance of palpable, chemically induced mammary tumors caused tumor regression (Table?4).

Administration of the nonaromatizable androgen drostanolone (2,10,13-trimethyl DHT) propionate (40?mg/kg, 5/week for 6?weeks) to rats had no effect on DMBA-induced or MCA-induced mammary tumors [16]. Yet both DMBA-induced and MCA-induced mammary tumors regressed following ovariectomy, demonstrating that the tumors remained hormone dependent. One explanation could be the dose dependency of androgen effects, as low dose (12 or 6?mg/kg weekly) induced tumor regression, consistent with another study using a different DHT analog (2?-methyl DHT) [49], whereas a very high TP dose (210?mg/kg weekly) stimulated DMBA-induced tumor growth [61]. These dose dependencies may reflect direct cross-reactivity of very high DHT doses with off-target estrogen or other receptor mechanisms.

#### Effect of anti-androgens or androgen receptor inactivation on mammary tumors

The anti-androgen flutamide has been used to investigate the role of androgens in chemical carcinogen-induced mammary tumors [76],[78]. Surprisingly, flutamide markedly inhibited the growth of DMBA-induced mammary tumors in female rats, contrasting with the inhibitory effect of androgens. The most probable mechanism is that flutamide has weak direct partial agonist effects on AR when sufficiently high flutamide doses are applied on a low androgenic background (for example, in female or castrated animals) [80]. Other less likely mechanisms include: inhibition of adrenal steroid production, which is implausible given the rodent adrenal?s negligible androgen output [81]; or reduced endogenous P4 secretion [78], due to neuroendocrine effects on pituitary and/or ovarian hormone secretion [82]. The uncertainties reflect the limitations of using solely pharmacological probes to define steroidal molecular mechanisms.

Using an androgen receptor knockout (ARKO) androgen-insensitive female mouse model (expressing a mutated, inactive AR) combined with DMBA chemical carcinogenesis, however, we demonstrated that the onset of palpable mammary tumors was significantly faster in ARKO females compared with wild-type female mice (median time, 22?weeks vs 34?weeks, respectively) [1], supporting the inhibitory role of AR-mediated androgen actions in mammary carcinogenesis. This observation predicts that otherwise healthy women who are heterozygous for the functional and inactive AR alleles (for example, mothers of children with androgen insensitivity) could also be at increased risk of breast cancer, although this prediction remains to be tested. This prediction is also supported by increased breast cancer susceptibility in women with reduced AR activity due to longer polymorphic CAG repeats [83]. The relationship between AR CAG repeats and breast cancer susceptibility could be further tested using a humanized mouse model where AR activity is modified [84] by replacing the rodent *Ar* gene with most of the human *Ar* exon 1 CAG repeat length variant [85]. Hence, investigating the relationship between AR CAG repeats and breast cancer susceptibility may provide direct insight into the relationship between AR activity and breast cancer susceptibility in humans. This relationship has been suspected [83],[86] but not experimentally explored.

#### Effect of aromatase inhibitors on mammary tumor growth

Aromatase inhibitors inhibit mammary tumor growth in rodents [78],[87],[88]. Aromatase inhibitors cause increases in circulating testosterone concentrations with decreased E_2_ concentrations in both rodents [78],[88] and women [89]. Although the protective role of aromatase inhibitors on breast cancer due to reduced E_2_ concentrations is well established, the role of the concomitant increases in serum testosterone concentration has usually been overlooked, and could be further explored using genetically modified models with altered aromatase expression [90]-[93]. However, not only may aromatase inhibitors increase circulating endogenous androgen concentrations, but some possess intrinsic androgenic activity by binding to and activating the AR [78],[87] ? highlighting the limitations of pharmacological blockade of steroidal mechanisms, where blockers or their metabolites may have off-target effects. The protective role of aromatase inhibitors in breast cancer could therefore also be due to direct androgenic effects. This is supported by the superior efficacy of the aromatase inhibitor letrozole over the ER blocker tamoxifen (lacking AR activation) [94]. Hence, the effectiveness of aromatase inhibitors as breast cancer treatment may involve their largely overlooked direct androgenic effects on tumors, an interpretation that warrants further evaluation.

### Androgens and hormonal carcinogenesis

Proof of the experimental induction of mammary cancer by the pro-estrogen E_1_ as well as E_2_ in the 1940s was a landmark in the investigation of hormone-dependent breast cancer [95]. In rats, co-administration of testosterone increased latency for the appearance of E_1_-induced palpable tumors, in a dose-dependent manner [96]. In addition, administration of testosterone to rats already bearing E_1_-induced mammary tumors decreased the tumor area [96],[97]. These findings further support a protective role of androgens in experimental carcinogenesis, despite the mechanism of tumor induction (carcinogen or hormonally induced). This implies either direct AR-mediated regulation of cell proliferation or perhaps similar mechanisms involving induction of experimental tumors.

In contrast, Noble demonstrated that simultaneous treatment with E_1_ and TP in Noble rats significantly increased the incidence of mammary tumors compared with E_1_ treatment alone [98], further supported by more recent studies [99]-[101], although high doses of testosterone also reduced the latency of E_2_-induced tumors [101]. Testosterone may act as an endogenous promoter in estrogen-induced mammary carcinogenesis via stimulation of paracrine secretion of growth factors in mammary glands or oncogenes in mammary cells, rather than through the stimulation of breast cell proliferation [101]. However, the reasons for the contradictory findings on the impact of testosterone on hormone-induced breast cancer remain unknown.

### Androgens and spontaneous rodent carcinogenesis

Specific rat and mouse lines that develop spontaneous mammary tumors have been used to investigate the role of androgens in the incidence of these tumors, as well as their growth as tumor transplants (Table?5). Growth of 80% of spontaneous mammary tumors was prevented by TP treatment [102], while testosterone or DHT treatment induced significant regression of transplanted tumors [103]. DHT administration to ovariectomized rats caused greater regression of tumor growth, suggesting a direct effect on tumor cells; however, a contributory effect of androgens in reducing ovarian hormone secretion due to negative hypothalamic feedback mechanisms could not be excluded [103].

**Table 5:** **Androgen effects on spontaneous tumor growth/incidence**

Reference	Mouse/rat	Carcinogen	Dose ^a^	Duration	Tumor growth/incidence
**Tumor growth (treatment after palpable tumors)**					
Heiman [102]	White rat^b^	Spontaneous/autotransplant	5 to 15?mg TP (total dose)	4 to 6?months	?
Huggins and Mainzer [103]	SD rat	Transplants	6?mg?T/DHT weekly	7?weeks	?
Nathanson and Andervont [30]	C3H mice	Spontaneous	7 to 17.5?mg TP weekly	4?weeks	No effect
**Tumor incidence (treatment before palpable tumors)**					
Jones [104]	C3H mice	Spontaneous	1.5?mg TP weekly (weeks 1 to 3); 1?mg TP weekly (weeks 4 to 45)	45?weeks	?
Nathanson and Andervont [30]	C3H mice	Spontaneous	1.5?mg TP weekly	At 4.5?months of age for 4?months	?
Lacassagne and Raynaud [105]	C3H mice	Spontaneous	4?mg TP weekly	Beginning a few days after birth	?
Loeser [106]	Strong A strain mice	Spontaneous	28 to 56?mg TP (total dose)	Implant	?
Schwartz [107]	C3H mice	Spontaneous	1,350?mg/kg DHEA weekly	5 to 29?weeks of age	?
Heiman [108]	RIII mice	Spontaneous	8 to 9?mg TP (total dose)	6 to 7?months of age for 8 to 16?months	?
Yanai and colleagues [109]	SHN mice	Spontaneous	1?mg DHT daily (prenatal) or 0.2?mg DHT daily (neonatal)	GD 12 to 15 (prenatal) or daily for 5?days after birth (neonatal)	? (neonatal)
Mori and colleagues [110]	BALB/cfC3H mice	Spontaneous	0.02 or 0.005?mg?T daily	Days 1 to 5 after birth	?

DHEA, dehydroepiandrosterone; DHT, dihydrotestosterone; GD, gestation day; SD, Sprague?Dawley; T, testosterone; TP, testosterone propionate. ^a^Some doses are shown as weekly doses calculated based on the dosing regime in the original article. ^b^Female rats/mice unless otherwise specified.

Most studies [30],[102],[104]-[108], but not all [109],[110], demonstrated that the treatment of mice with TP or the pro-androgen DHEA before the development of palpable tumors reduced the incidence of tumors in mice (mainly C3H). In contrast, postnatal treatment (days 1 to 5) of SHN or BALB/cfC3H female mice with DHT or testosterone significantly increased mammary tumor incidence when compared with vehicle-treated control groups [109],[110]. These opposite effects could be related to timing of the androgen exposure, but the significance is still uncertain.

### Androgens and genetically modified mouse breast cancer models

In our studies, androgen-insensitive female mice (global ARKO model) have increased susceptibility to DMBA-induced mammary tumors, which demonstrates direct mammary gland-specific effects of androgens acting via the AR [1]. A study using Cre/LoxP-mediated conditional deletion of the *Ar* allele in mammary epithelium demonstrated that reduced epithelial AR expression also promoted the early onset of Neu (ERBB2) proto-oncogene tumors [32], suggesting that the AR in mammary epithelial cells may have a significant role in AR-dependent protection against experimental breast cancer. Yet transgenic rats overexpressing rat ERBB2 in the mammary gland developed mammary tumors only in males and not in females, suggesting that the development of tumors was androgen dependent [111]. This androgen dependency was further supported by findings that orchidectomy prevented male mammary tumor development while DHT treatment restored it. This discrepancy between mouse and rat models targeting the ERBB2 pathway may be due to the fact that the mouse model overexpressed the mutated form of ERBB2 [32], whereas the rat model overexpressed nonmutated ERBB2 [111] and rat tumors were strongly AR-positive while mouse tumors had low AR immunopositivity. While only few studies have so far utilized the ARKO mouse models, in the future these models (both global and cell-specific ARKO) combined with various transgenic experimental breast cancer models may be uniquely enlightening when exploring the cell-specific role of AR as well as the influence of AR-mediated androgen actions on specific carcinogenic pathways.

## Discussion and conclusions

Rodent models have been used to investigate hormonal mechanisms in experimental breast cancer, but the lack of uniformity of findings ? in terms of experimental design, species, strain, mechanism of tumor induction, and types of androgens used ? renders it difficult to develop an integrated explanatory mechanism. Yet, despite considerable variability in study design, most rodent studies suggest a protective role for androgens at several stages of experimental breast cancer. Nevertheless, a minority suggests that androgens increase, or anti-androgens protect against, experimental breast cancer. The reasons for these discrepancies may provide potentially important clues to unravel the role of androgens in experimental breast cancer. The suggested explanatory framework involves a biphasic effect of androgens with low or moderate doses inducing tumor regression, while higher doses have no or stimulatory effects on tumor growth. The growth-promoting effect at high androgen doses could be via ER-dependent mechanisms: either via enzymatic conversion of aromatizable androgens used or via direct binding of supraphysiological androgen concentrations to the ER.

AR-mediated androgen signaling has recently been suggested to be inhibitory in ER?-positive breast cancers, opposing the effect of estrogens, while androgens in the presence of AR may activate the oncogenic properties of AR in ER?-negative tumors (reviewed in [2]). This concept is also utilized in the clinical setting, where AR inhibition is considered a potential therapeutic target for ER?-negative but not AR-positive breast cancers. The influence of androgens on experimentally induced ER?-positive or ER?-negative cancers has not been systematically evaluated in rodent studies. This is possibly due to the difficulty in developing estrogen-dependent, ER?-positive tumors in mice. DMBA has been shown to induce the development of ER?-positive tumors in rats [74] and mice [1], and thus could allow for the comparison of tumor progression between ER?-positive and ER?-negative tumors. Similarly, while ER? status is usually analyzed in experimental breast cancer models, AR expression has not been systematically determined. Much more in-depth analysis of AR expression and the role of AR-mediated androgen action as well as modifying factors is therefore necessary to optimize the opportunity afforded by the role of androgen action at all stages of experimental breast cancer.

These opportunities are being developed with therapies targeted to AR or androgen production involved in recently initiated, empirically motivated clinical trials [112]-[114]. Interestingly, the anti-androgen bicalutamide showed some treatment efficacy in a phase II clinical trial testing treatment for ER-negative/AR-positive breast cancers (ClinicalTrials.gov NCT00468715) [112]. However, as discussed above regarding anti-androgens in experimental breast cancer, anti-androgens such as bicalutamide can also have agonist properties, demonstrated clinically by a decline in serum prostate-specific antigen following bicalutamide withdrawal [115]. Largely, the mechanisms of androgen actions as well as the modifying factors involved are still not sufficiently well known. More detailed mechanistically oriented experimental work on androgen metabolism, on the mechanisms of androgen action, as well as on modifying/interacting factors for the role of the AR in breast cancer is therefore still needed in order to develop efficient and better targeted androgen therapies.

In conclusion, while androgens are assumed inhibitory and estrogens stimulatory for mammary cell proliferation in experimental settings, it is possible that the ratios of these hormones and/or their respective receptors may regulate the optimal balance in tissue maintenance and if disturbed will lead to pathological/malignant growth. As the AR is one of the most abundant steroid receptors present in breast cancers, more research is warranted to understand androgen actions via the AR in breast tissue as a regulator for optimal hormonal balance and to provide opportunities for novel therapeutic targets and prognostic biomarkers for this lethal disease.

## Abbreviations

AR: Androgen receptor
ARKO: Androgen receptor knockout
CYP: Cytochrome P450
DHEA: Dehydroepiandrosterone
DHT: Dihydrotestosterone
DMBA: 7,12-dimethylbenz(a)anthracene
E_1_: Estrone
E_2_: Estradiol
ER: Estrogen receptor
MCA: Methylcholanthrene
TP: Testosterone propionate
